# Resident and staff perceptions of an activity- and recovery-based intervention in supported housing for people with severe mental illness – a longitudinal pilot study

**DOI:** 10.1186/s12888-022-04050-7

**Published:** 2022-06-16

**Authors:** Mona Eklund, Carina Tjörnstrand

**Affiliations:** Department of Health Sciences, Lund University, P. O. Box 157, SE-22100 Lund, Sweden

**Keywords:** Activity, Intervention, Occupational therapy, Psychiatric disabilities, Supported accommodation, Recovery

## Abstract

**Background:**

People with severe mental illness who reside in supported housing (SH) and need a high level of assistance are at risk of an everyday life with little meaning and low community participation. Interventions to counteract that seem warranted, which was the rationale for this study. The aim was to investigate how residents and staff perceived an intervention designed to enhance meaningful everyday activity and personal recovery.

**Methods:**

The intervention, termed Active in My Home (AiMH), was led by an occupational therapist. It consisted of five individual and three group sessions, and AiMH staff acted as supporters. Twenty-nine AiMH participants and 43 staff members were included in this un-controlled study with three measurement points – before (T1), at completion (T2), and 6–9 months after completion of AiMH (T3). The data collection was based on self-report questionnaires addressing perceptions of satisfaction, meaningfulness, and recovery-oriented support.

**Results:**

The residents’ satisfaction with the SH per se was rated high (at 75% of the maximum score) and did not change over the study period from T1 to T3 (*p* = 0.544); nor did the participants’ perceived recovery-oriented support from the AiMH supporter (*p* = 0.235). Satisfaction with AiMH was rated by both participants and staff at T2. Their scores differed regarding general satisfaction (*p* = 0.008), staff scoring higher, but no differences were found regarding satisfaction with group sessions, individual sessions, or support of activity (*p*-values 0.062–0.836). The staff rated the SH unit’s provision of meaningful activities higher than the AIMH participants at T2 (*p* = 0.029) but not at T1 (*p* = 0.226) or T3 (*p* = 0.499).

**Conclusion:**

This study has offered some glimpses of how AiMH participants and staff perceived the AiMH intervention. It has also generated some ideas for better support for meaningful activity and recovery-oriented support in SH for people with mental illness, such as assisting SH residents in identifying activity opportunities and making activity choices when providing support for meaningful activity in the SH context.

**Trial registration:**

Registered at ClinicalTrials.gov ID: NCT05157854.

## Introduction

People with severe mental illness who reside in supported housing (SH) tend to have unmet needs concerning social relations and daily activities [1, 2]. They are at risk of an everyday life with little meaning and activity within the SH premises and little participation in the community, as evidenced by both SH residents [3] and their informal carers [4]. However, differences in staff location, level of support, degree of stress on moving on, and type of physical setting create an abundance in types of SH settings, as acknowledged by McPherson and colleagues when categorizing SH units [5]. These authors proposed a classification (Simple Taxonomy for Supported Accommodation [STAX-SA]), and in the current study SH is defined according to their Type 1, with staff continuously on-site, a high degree of support, little emphasis on moving to a more independent type of accommodation, and a congregate physical setting. Residents in this type of high-support settings have reported about lack of friendship but forced togetherness, and few opportunities for self-chosen activities [3]. Informal carers described that their dear ones residing in SH needed continuous support, including transports, to uphold a hobby or an interest [4].

Although research in the SH context is not uncommon, interventions to counteract meaninglessness in everyday life and low community participation seem scarce. Bitter and colleagues [1] reviewed the literature for recovery-oriented support in the context of SH and other long-term inpatient psychiatric services. They used wide selection criteria and included not only SH according to the STAX-SA classification, but also rehabilitation and treatment settings. By their wide criteria, they identifies 53 studies, and 11 of these included SH settings only. Six of these showed some type of recovery-oriented positive outcome, but none of the 11 studies addressed meaningful activity, otherwise seen as an important aspect of personal recovery [6]. The scarcity of SH interventions targeting a meaningful and active everyday life made Eklund et al. [7] develop the eight-session Active in my Home (AiMH) intervention for use in SH in Sweden. In order to test the new intervention, they developed a pilot and feasibility study with no control and found that the AiMH participants increased their engagement in activities and improved their personal recovery from baseline to completed intervention [7].

Since meaningful activity has been found to be consistently associated with better well-being and quality of life among people with mental illness [8–10] and to enhance personal recovery [11], the findings of few and predominantly quiet activities in the SH context [3, 4, 12] prompted the development and evaluation of the AiMH intervention [7, 13]. The present longitudinal pilot study continues the reporting from the AiMH project. The aim was to investigate residents’ and staff’s perceptions of support for activity and recovery in the SH unit, with a specific focus on the AiMH intervention. The following research questions guided the study:

- How did residents rate their satisfaction with the SH services over time? And how was that related to their satisfaction with the AiMH intervention?
- How did SH staff rate their satisfaction with the AiMH intervention? Did their perception differ from that of the residents?
- How did residents and staff rate the SH unit’s provision of meaningful activities?
- How did residents rate recovery-oriented support from their mental health workers?

## Methods

The longitudinal design of this study involved three measurement points – at baseline before the start of the AiMH intervention (T1), after completed AiMH (T2), and a follow-up 6–9 months after completed AiMH (T3). The Regional Ethics Committee at Lund University approved the study (reg. no. LU-2015/873).

### The AiMH intervention

A detailed description of the AiMH intervention, framed as a course, can be found elsewhere [7, 13]. It builds on a frame of reference based on five compatible lines of reasoning around functioning and well-being, here termed cornerstones. One is *occupational therapy*, maintaining that being active is a human need and promotes health and well-being [14]. *Personal recovery*, which is an individual process of regaining a meaningful and satisfying life despite mental illness [15], forms a second cornerstone, and *International Classification of Functioning* (ICF) [16] a third. ICF defines disability as the result of an interaction between the individual and the environment and highlights how the environment can facilitate or hinder the individual’s participation in society. The fourth cornerstone is *sensory modulation*, which sets focus on individuals’ sensory systems. Sensory inputs can be either calming or arousing and can be modulated to fit the individual’s current needs and mental state [17]. The final cornerstone is the *remotivation process* [18], which is about how to stimulate and maintain an individual’s motivation for activity.

AiMH starts with two workshops for staff, to introduce them to the intervention and the contributions expected from them. Their main task is to act as personal supporters for the AiMH participants, during sessions and when testing activities in real life. Video-recordings and written material from the workshops were provided as extra support for staff and to offer new staff the chance to catch up. AiMH consists of eight 30–45 minute sessions for the residents, five individual ones and three in a group. Each session has a theme, such as: “activity and health”, and “what are my dreams – and how can I realize them?” The sessions are built up with short lessons and exercises, using one’s senses such as smell or touch, testing a simple activity, and setting goals for an activity one wants to test between sessions. Experiences from exercises and between-session activities are shared and discussed to the extent that the participants want. The participants are given around 20 EUR to try out that activity, preferably in the community. The AiMH intervention as a whole is distributed over about twelve weeks.

The role of the AiMH supporters includes participating in a couple of the sessions, inspiring the course participant to stick to strategies developed during the sessions and supporting activity in general between the occupational therapy sessions.

AiMH exists in two versions, with the same contents but slightly different structure. The pilot version (AiMH 1.0) [13] had a stronger emphasis on groups (three individual sessions and five group sessions), compared to the current version (AiMH 2.0) where five are individual and three are group sessions. Although the session themes are the same for both versions, AiMH 2.0 offers more varied levels of difficulty to enable adjustment to the participants’ capacities. The current study is based mainly on AiMH 2.0, and further explanation is found in the description of recruitment of AiMH participants.

### Study context

The study was performed in urban and rural areas in southern Sweden. The SH units were congregate settings where residents had a room or small apartment. They had access to communal areas for meals and company and, for between 12 to 24 hours per day, support from staff. The occupational therapists who gave the AiMH course were employed by the research team, as the occupational therapists who were employed in the included municipalities only had a consulting role vis-à-vis staff and did not work directly with SH residents.

### Recruitment of SH units

Recruitment of SH units occurred in two steps. First, municipalities in southern Sweden were strategically selected based on urban/rural variation. Second, variation was sought among the SH units regarding socioeconomic situation and proportion of immigrants in their catchment areas, as well as on size of the SH units. After consent from managers in five municipalities, representatives from fifteen SH units were approached and received information about the project. Seven agreed to participate. Motivations for declining included fearing that the AiMH course would be too difficult for the residents. This two-step procedure resulted in four municipalities contributing with one unit each and the fifth (a medium-sized city) contributing with three.

### Selection of participants

In order to recruit AiMH participants, a member of the research team and an occupational therapist held an information meeting at each involved SH unit. The residents received both oral and written information, which included the nature of the study and what participation would be expected if they consented. They were also informed that participation was voluntary and that they could decline further participation at any time. This resulted in 29 residents who gave their written informed consent. Seven of these had participated in AiMH 1.0, the remaining 22 in AiMH 2.0. No-one had participated in both. Since there was no substantial difference between the original AiMH 1.0 and the somewhat developed AiMH 2.0, we chose to include all 29 participants in the current study to increase statistical power.

Recruitment of staff, to become AiMH supporters as well as study participants, took place simultaneously with the recruitment of residents. In all 43 staff members agreed to participate and gave written informed consent. Thirty-four of these participated from the start, and the others joined later as a consequence of staff turnover. Who were individual AiMH supporters and who functioned as team members in general was not reported.

### Data collection

The data collection consisted of a background questionnaire, self-report instruments, and assessments made by the research assistant who administered the data collection. Brief versions of instruments were used when available, to avoid a risk of stress and discomfort among AiMH participants in connection with the data collection.

The background questionnaire was devised for this project and included socio-demographic data and self-reported diagnosis. The diagnoses were subsequently grouped by the research team into ‘psychosis’ and ‘other’, which included neuropsychiatric disorders and borderline personality disorder.

#### Satisfaction with the SH services

The housing satisfaction questionnaire (HSQ) is tailored for SH residents and encourages them to think about various aspects of their accommodation, their physical home as well as the support and help they receive there [7]. The HSQ has eight items that are rated on a four-point scale from one to four, where a higher score reflects greater satisfaction. The maximum score is 32. Sample items are “Do you have the type of housing you want?” and “Would you recommend your housing to a friend?” HSQ is not widely used but has shown good internal consistency reliability [7], also based on the present sample (α = 0.82).

#### Satisfaction with the AiMH intervention

Based on the HSQ described above, two separate satisfaction scales were developed to assess satisfaction with the AiMH intervention, one for AiMH participants and one for staff. The AiMH participant version has seven items and corresponds closely to the HSQ, just slightly reworded to specify the intervention. They are rated on a four-point scale from one to four, and the maximum score is 28. Internal consistency reliability was α = 0.93.

The staff version was more comprehensively elaborated, and only four of the HSQ original items were retained, somewhat reformulated to fit the staff perspective. Added items addressed, e.g., the quality of the workshops, perceived support from the occupational therapist, and whether AiMH had brought any inspiration to the work in the SH. The response format was the same as for AiMH participants. This staff version did not produce a scale with sufficient internal consistency reliability, nor did it when reduced to the four items that corresponded to the AiMH participant version. It was therefore decided to analyze these four items separately when used in calculations including staff. They concerned the respondent’s view of the quality of the individual sessions, the group sessions, and the support for activity, as well as general satisfaction with the intervention.

#### Provision of meaningful activities

The seven-item version of Perceived Meaning of Activity in Housing (PMA-H-7) [19] was used to assess AiMH participants’ and staff’s perceptions of the SH unit’s provision of meaningful activities for the residents. The items are formulated as statements, preceded by the anchor “My housing contributes to …” . A psychometric study has indicated acceptable internal consistency (α = 0.75) and construct validity in terms of convergent and discriminant validity [19]. Internal consistency reliability based on the current sample was α = 0.76 for the AiMH participants and α = 0.79 for the staff.

#### Recovery-oriented staff support

Brief Inspire is a five-item rating scale where a person with mental illness can rate the support they receive from their mental health worker. It has shown to form a single factor and internal consistency and test-retest reliability [20]. Sample items are “My worker helps me to have hopes and dreams for the future” and “My worker helps me to feel good about myself”. A five-point rating scale is used in the current study with response alternatives varying from “not at all” (=1) to “very much” (=5). This makes a possible top score of 25, indicating maximum support for recovery. Brief Inspire is a short version of the original Inspire, which has previously been translated into Swedish and been found to have good face and content validity and satisfactory internal consistency [21]. Since there was no version of Brief Inspire available in Swedish, our research team made a translation and back-translation. Internal consistency reliability calculated on the current sample was adequate at α = 0.78.

The data collection was carried out on three occasions – at baseline (T1), after about 12 weeks at completed AiMH (T2), and at a follow-up after 6–9 months (T3). AiMH participants’ and staff’s satisfaction with the AiMH intervention was assessed only at T2, but all other instruments were completed on all three occasions. Research assistants who were not part of the research team administered the data collection. They were all occupational therapists with experience of working in mental health care and as research assistants. When needed, they could assist the AiMH participants by explaining questions or reading out loud, but they were careful not to influence the answers in any direction.

### Data analysis

The data were analyzed by non-parametric statistics; Spearman correlations to calculate relationships between variables, the Mann-Whitney test to compare groups, and Friedman’s test to address changes over time. Estimations of the strength of correlations were based on Cohen [22], who proposed that correlations < 0.30 are weak, 0.30–0.50 are moderate and > 0.50 are strong. Change scores of interest were those based on T1 and T2. They were calculated by subtracting the T1 score from the T2 score. A *p*-value of < 0.05 was set for statistical significance. Since the study had few participants, entailing a risk of type-2 errors [23], a limit for non-significance was set as well, at *p* > 0.1. The software used for these analyses was SPSS 26.0 [24].

## Results

### Presentation of participants

More than half of the AiMH participants were men, all who responded to the question regarding civil status reported being single, and the most commonly self-reported diagnosis was psychosis. Four of the participants had lived in their current accommodation for less than a year, and eight had lived there for 10 years or more. The participant who had lived there the longest had been a resident there for 26 years. Further characteristics are presented in Table 1.

**Table 1:** Characteristics of the 29 AiMH participants

*Characteristic*	*Mean (SD) or nos.*
Age; mean (SD)	47 (13)
Gender; men/women (nos.)	18/11
Civil status; single/missing data (nos.)	27/2
Born in Sweden; yes/no (nos.)	25/4
Highest education level; 9-year school/high school/university (nos.)	11/15/3
Self-reported diagnosis; psychosis/other/missing data (nos.)	17/7/5
Years in current accommodation; mean (SD)	7.6 (5.8)
Psychosocial functioning (GAF) at baseline; mean (SD)	47.6 (8.5)
Psychiatric symptoms (GAF) at baseline; mean (SD)	45.6 (11.5)

Participating front-line staff varied over the three occasions for data collection. They were 43 in total; 34 responded at T1, 23 at T2, and 9 at T3. Twelve participated in both T1 and T2, and five in T1, T2 and T3. Their mean age (SD) was 45 (13) years and 79% were women. Twenty-seven of them (63%) were educated as nurse assistants, mostly with specialization in mental health care. The remaining 16 participants from front-line staff had education varying from high school only to a PhD degree; 10 of them (23% of the total sample) had a university degree. Their experience of working within mental health care or support varied from 7 months to 44 years, with a mean of (SD of) of 13 (12) years.

### Satisfaction with housing and the AiMH intervention

The residents rated their satisfaction with housing at T1, T2 and T3. Their mean ratings (SD) for the sum score were 24.3 (4.6) at T1, 23.7 (4.9) at T2 and 24.7 (5) at T3. These ratings were stable over time; no difference could be discerned (chi2 = 1.22, *p* = 0.544). Upon completed intervention, at T2, the residents also rated their satisfaction with AiMH and scored on average 22.9 (4.0). The correlation with the satisfaction with housing rating performed on the same occasion was r_s_ = 0.56 (*p* = 0.003). As shown in Table 2, there was no statistically significant difference between the residents’ and the staff’s ratings on the separate items addressing the individual sessions (Z = -0.207), the group sessions (Z = -1.865), or support for activity (Z = -0.840). There was a difference on the item addressing general satisfaction with AiMH, however, staff scoring higher (Z = -2.638).

**Table 2:** AiMH participants’ and staff’s satisfaction with the intervention at T2; mean (SD)

	Participants	Staff	P-value
Satisfaction with individual sessions	3.4 (0.6)	3.5 (0.5)	0.836
Satisfaction with group sessions	3.2 (0.6)	3.5 (0.5)	0.062
Satisfaction with support for activity	3.2 (0.6)	3 (0.7)	0.402
General AiMH satisfaction	3.3 (0.7)	3.8 (0.4)	0.008

### Perceptions of the SH unit’s provision of meaningful activities

Residents and staff rated at T1, T2 and T3 how they viewed the provision of meaningful activities in the SH unit. The residents’ ratings (SD) were 24.2 (6), 23.5 (6.4) and 23.5 (7.1) and these ratings were stable over time (chi2 = 0.88, *p* = 0.646). The staff ratings were 26.1 (4.3), 27.1 (3.7) and 25.2 (4.4), also stable over time (chi2 = 2, *p* = 0.368). There was a statistically significant difference at T2 (Z = -2.28, *p* = 0.029), staff rating the unit as providing more meaningful activities compared to the residents. No differences were found at T1 (*p* = 0.226) and T3 (*p* = 0.499).

### Residents’ perceived recovery-oriented support and associations with AiMH satisfaction ratings

Self-reports regarding the residents’ perceived general recovery-oriented support were stable over time (*p* = 0.235). The scores were 18.3 (3.5) at T1, 17.7 (4.6) at T2, and 19.8 (4.9) at T3. Their satisfaction with the AiMH intervention, measured at T2, was not associated with their perceived general support towards recovery at that time (*p* = 0.813) or to change in perceived support from T1 to T2 (*p* = 0.411). There was a statistically significant correlation between the AiMH participants’ perceived recovery-oriented support and satisfaction with the SH unit at T1 (r_s_ = 0.42, *p* = 0.025), but not at T2 (*p* = 0.590) or T3 (*p* = 0.051).

## Discussion

This study has shed some light on AiMH participants’ and staff’s view of the AiMH intervention for people with mental illness living in SH, as well as selected general qualities of their SH unit.

### General SH qualities

The general SH qualities addressed were the AiMH participants’ perceived housing satisfaction, AiMH participants’ and staff’s views of the SH unit’s provision of meaningful activity, and the AiMH participants’ perceived recovery-oriented support. The AiMH participants’ mean score in housing satisfaction was around 24 on all occasions (75% of the maximum score). This must be regarded as indicating a high level of satisfaction with received support, a common finding in mental health care and SH settings [25], and the scores were close to identical with a cross-sectional study in the SH context [7]. The fact that housing satisfaction was stable over time indicates that the AiMH intervention did not affect housing satisfaction. One needs to bear in mind, however, that when satisfaction levels are already high at baseline it is hard to achieve improvements. This may have been further accentuated by possible selection bias. It became clear during recruitment of SH units that managers found the intervention to be on the difficult side in relation to their residents, and it is conceivable that those who were most afflicted by their mental illness were not invited to AiMH. Compared to a larger sample of Swedish SH residents, participating in a descriptive study [7], the current sample was similar on most known characteristics. Regarding educational background, however, a larger proportion in the current sample had high school as their highest education and a smaller proportion had completed only 9-year school or lower. It is also important to consider the possibility of regression to the mean, which appears when baseline data are very high, in turn entailing that a second measurement is likely to result in a score that is closer to the mean [26].

The findings regarding the SH unit’s provision of meaningful activities showed that both AiMH participants’ and staff’s ratings were stable over time. Interestingly, at T2, when the AiMH intervention had just been completed, the staff scored significantly higher than the AiMH participants on the unit’s assortment of meaningful things to do. This may suggest that the staff had a stronger focus on the efforts they had made to enrich the SH activities and identified more opportunities for meaningful activity compared to the AiMH participants.

The AiMH participants’ perceived recovery-oriented support from the staff was stable over time and corresponded to 70–80% of the maximum score. Recovery-oriented support was not associated with the AiMH participants’ satisfaction with the intervention, measured only at T2. At T1 there was a medium-sized correlation with satisfaction with the SH unit, however, suggesting that the SH support in general and the recovery-oriented support were partly over-lapping phenomena. The finding that no corresponding correlation with satisfaction with the SH unit was seen at T2, after having completed AiMH and experienced new forms for support, suggests that the AiMH intervention may have had some unknown effect on how the participants viewed the character of the received support. Staff turnover could also be an influential factor; some participants had lost their initial AiMH supporters at that time. Although video-recordings and written material were provided for new staff, the entire experience of being introduced to being an AiMH supporter could not be reproduced. Qualitative research would be important to generate deeper knowledge about possible impact of staff turnover on the participants’ experiences.

### Opinions on the AiMH intervention

AiMH participants rated their satisfaction with the intervention high after completion, at 82% of the attainable score. The association between satisfaction with general SH qualities and satisfaction with the AiMH intervention was in the realm of strong [22]. Had there been an increase in satisfaction with the general qualities of the SH unit from T1 to T2, a strong correlation with AiMH satisfaction at T2 could have been regarded as a sign of a successful intervention. However, since the satisfaction with the SH unit’s general qualities was stable over time, this association with AiMH satisfaction at T2 suggests nothing about any added value of the intervention. A more likely conclusion is that both ratings reflected a common satisfaction tendency, in line with what has been proposed by Priebe and colleagues [27]. Importantly, however, a related study found a relationship between satisfaction with AiMH and increased activity engagement after completed intervention [7, 28], which might indicate that AiMH is an adequate SH intervention to promote activity engagement.

With respect to the four items that were analyzed from both AiMH participants’ and staff’s perspectives, the staff rated their general satisfaction higher. There was also a tendency that they were more satisfied than the AiMH participants with the group sessions. These signs of greater satisfaction in the staff group may indicate that the staff assigned a higher value to the intervention than did the participants, also corroborated by the finding of higher ratings from staff regarding the SH unit’s provision of meaningful activities after completed intervention. That staff show greater satisfaction than clients with activity-based methods is not unique. A parallel can be drawn to findings from a study where staff found an activity-based assessment method better than standard procedures, but the same was not seen from the perspective of clients with a mental illness [29, 30]. It is possible that when a new method is launched in the service provision, shared by the team as a whole, they find they have something new in common, and as a result their professional identity is enhanced.

### Implications for support for activity in the SH context

Both AiMH participants and staff rated their satisfaction with the intervention high, which suggests that AiMH is suitable for the SH context. Still, the fact that the staff were generally more positive towards the intervention than the participants suggests that working further with prospective participants’ motivation, possibly as preparation before the AiMH start, might be a good strategy. The sum of 20 EUR for participants to spend on activities did probably not work as an incentive, but rather made the AiMH viable. There was also an indication, based on a *p*-value < 0.1, that the participants might be less apt to appreciate the group sessions. Research has shown that people with mental illness may hesitate to join a group, but once accustomed to being in a group they benefit greatly and view the group as essential in their journey towards meaningful activity and recovery [31, 32]. Preparing participants for being in a group, along with motivational work, would thus be an additional important strategy for support for activity among SH residents. Findings also indicated that, at completed AiMH, the staff seemed to have identified more opportunities for meaningful activity compared to the AiMH participants. Pedagogical methods, such as discussions, positive feedback, information and brainstorming, which may be seen as aspects of a remotivation process [18], might assist SH residents in identifying opportunities for meaningful activity they may otherwise overlook. That would facilitate choice for the residents, which has been found to be central for recovery among people with mental illness living in SH [33]. In the endeavor to increase activity opportunities for residents in SH it is however important to consider that, compared to those with mental illness who live in ordinary housing, SH residents tend to be more satisfied with a low level of activity [7].

Another way of boosting AiMH as an intervention in SH could be to concomitantly train staff in recovery-oriented support. Previous research has found such training successful. The effect levelled out somewhat over time [34], indicating that such training should be part of continuous staff education. A third alternative could be to apply the principle of place-then-train, used in work rehabilitation according to the Individual Placement and Support model [35]. Transformed to the AiMH context, and if resources are available, this could imply that an AiMH supporter is at hand as the participant regularly participates in a self-chosen activity in the community, gradually getting more autonomous and self-going.

### Methodological considerations

This pilot study did not include a comparison group, since recruitment was highly problematic. This is in line with the circumstances described by Killaspy and colleagues [36] and is something that deserves further attention in research addressing the study context per se. Despite using all recruited SH units and participants for the intervention arm, the study was still underpowered. There is thus a risk for Type-2 errors, that true differences and associations have gone undetected. This is why a limit was set also for statistical non-significance (*p* > 0.1). The small sample size, particularly at the follow-up, was also the reason for the choice of statistical methods, avoiding a repeated-measurements approach. The small format weakens both internal and external validity of the study, which still has some value since it sheds some light on a poorly researched area of great importance for the quality of life of people with severe mental illness and a great need of support. The study has also given rise to further research ideas, such as understanding the role of AiMH in the general recovery-oriented SH support and identifying the obstacles that seem to be inherent in the SH context for intervention research.

## Conclusion

The AiMH was appreciated by both participants and staff, but the staff reported somewhat higher satisfaction than residents, and the staff perceived themselves to be providing more meaningful activities than residents perceived. This indicates that the intervention could be further optimized. A combination of motivational work, careful preparation for being in a group and pedagogical methods to assist SH residents in identifying activity opportunities and making choices may be useful strategies when providing support for meaningful activity in the SH context. This might increase the participants’ perceived gains from the intervention. Staff training to promote recovery-oriented support and using place-then-train principles to support community participation would be other ways of boosting the gains of AiMH. This small-scale study has offered some glimpses of how AiMH participants and staff perceived the AiMH intervention. It has also generated some ideas for future research that can lead to better support for meaningful activity and recovery-oriented support in SH for people with mental illness.

## Data Availability

The datasets generated and analyzed during the current study are not publicly available due to the Swedish Act (2003:460) concerning the Ethical Review of Research Involving Humans, but are available from the corresponding author on reasonable request.

## Abbreviations

AiMH: Active in My Home
SH: Supported housing
T1: At baseline
T2: After completed AiMH
T3: At a follow-up after 6–9 months

## References

[1] Bitter N, Roeg D, van Nieuwenhuizen C, van Weeghel J (2020). Recovery in supported accommodations: a scoping review and synthesis of interventions for people with severe mental illness. Community Ment Health J.

[2] Bitter NA, Roeg DP, van Nieuwenhuizen C, van Weeghel J (2016). Identifying profiles of service users in housing services and exploring their quality of life and care needs. BMC Psychiatry.

[3] Tjörnstrand C, Eklund M, Bejerholm U, Argentzell E, Brunt D (2020). A day in the life of people with severe mental illness living in supported housing. BMC Psychiatry.

[4] Gunnarsson AB, Brunt D, Tjornstrand C, Argentzell E, Bejerholm U, Eklund M (2020). Navigating in a misty landscape - perceptions of supporting a relative residing in supported housing for people with a psychiatric disability. Issues Ment Health Nurs.

[5] McPherson P, Krotofil J, Killaspy H. What works? Toward a new classification system for mental health supported accommodation services: the simple taxonomy for supported accommodation (STAX-SA). Int J Environ Res Public Health. 2018. 10.3390/ijerph15020190. 10.3390/ijerph15020190 PMC5858263 29364171

[6] Le Boutillier C, Leamy M, Bird VJ, Davidson L, Williams J, Slade M (2011). What does recovery mean in practice? A qualitative analysis of international recovery-oriented practice guidance. Psychiatr Serv.

[7] Eklund M, Argentzell E, Bejerholm U, Tjörnstrand C, Brunt D (2017). Wellbeing, activity and housing satisfaction - comparing residents with psychiatric disabilities in supported housing and ordinary housing with support. BMC Psychiatry..

[8] Aubin G, Hachey R, Mercier C (1999). Meaning of daily activities and subjective quality of life in people with severe mental illness. Scand J Occup Ther.

[9] Aubin G, Hachey R, Mercier C (2002). The significance of daily activities in persons with severe mental disorders. Can J Occup Ther.

[10] Eklund M, Hansson L, Bejerholm U (2001). Relationships between satisfaction with occupational factors and health-related variables in schizophrenia outpatients. Soc Psychiatry Psychiatr Epidemiol.

[11] Argentzell E, Bäckström M, Lund K, Eklund M (2020). Exploring mediators of the recovery process over time among mental health service users, using a mixed model regression analysis based on cluster RCT data. BMC Psychiatry.

[12] Bejerholm U, Eklund M (2004). Time-use and occupational performance among persons with schizophrenia. Occup Ther Ment Health.

[13] Tjörnstrand C, Argentzell E, Bejerholm U, Brunt D, Eklund M (2017). Active in My Home (AiMH) – a pilot and feasibility study of a lifestyle intervention in supported housing for people with psychiatric disabilities.

[14] Wilcock AA, Hocking C (2015). An occupational perspective of health 3ed.

[15] Anthony W (1993). Recovery from mental illness: the guiding vision of the mental health service system in the 1990s. Psychosoc Rehab J.

[16] WHO (2001). International classification of functioning, disability and health.

[17] Champagne T (2011). Sensory modulation & environment: essential elements of occupation.

[18] de las Heras CG, Llerena V, Kielhofner G (2003). Remotivation Process: Progressive intervention for individuals with severe volitional challenges (Version 1.0). Swedish version 1.0 (2012) by Wahlström Wärngård G.

[19] Eklund M, Brunt D. Development of 7-item perceived meaning of activity in housing (PMA-H-7) to assess opportunities for meaningful activities in the supported housing context for people with psychiatric disabilities. Eval Health Prof. 2019. 10.1177/0163278719845036. 10.1177/0163278719845036 31060380

[20] Williams J, Leamy M, Bird V, Le Boutillier C, Norton S, Pesola F (2015). Development and evaluation of the INSPIRE measure of staff support for personal recovery. Soc Psychiatry Psychiatr Epidemiol.

[21] Schön UK, Svedberg P, Rosenberg D (2015). Evaluating the INSPIRE measure of staff support for personal recovery in a Swedish psychiatric context. Nord J Psychiatry.

[22] Cohen J (1988). Statistical power analysis for the behavioral sciences.

[23] Altman DG (1993). Practical statistics for medical research.

[24] IBM SPSS Statistics 26 core system user’s guide Armonk [NY]: IBM Corp.; 2019.ftp://public.dhe.ibm.com/software/analytics/spss/documentation/statistics/26.0/en/client/Manuals/IBM_SPSS_Statistics_Core_System_User_Guide.pdf. Accessed 15 Nov 2021.

[25] Brolin R, Rask M, Syren S, Baigi A, Brunt DA (2015). Satisfaction with housing and housing support for people with psychiatric disabilities. Issues Ment Health Nurs.

[26] Chiolero A, Paradis G, Rich B, Hanley JA (2013). Assessing the relationship between the baseline value of a continuous variable and subsequent change over time. Front Public Health.

[27] Priebe S, Kaiser W, Huxley PJ, Roder-Wanner UU, Rudolf H (1998). Do different subjective evaluation criteria reflect distinct constructs?. J Nerv Ment Dis.

[28] Eklund M, Argentzell E, Bejerholm U, Brunt D, Tjörnstrand C (2021). Corrigendum. Br J Occup Ther.

[29] Kjellin L, Sjödahl RC, Eklund M (2008). Activity-based assessment (BIA)--inter-rater reliability and staff experiences. Scand J Occup Ther.

[30] Eklund M, Örnsberg L, Ekström C, Jansson B, Kjellin L (2008). Outcomes of activity-based assessment (BIA) compared with standard assessment in occupational therapy. Scand J Occup Ther.

[31] Eklund M (1997). Therapeutic factors in occupational group therapy identified by patients discharged from a psychiatric day center and their significant others. Occup Ther Int.

[32] Lund K, Argentzell E, Leufstadius C, Tjörnstrand C, Eklund M (2019). Joining, belonging, and re-valuing: a process of meaning-making through group participation in a mental health lifestyle intervention. Scand J Occup Ther.

[33] Piat M, Seida K, Padgett D (2019). Choice and personal recovery for people with serious mental illness living in supported housing. J Ment Health.

[34] Bitter N, Roeg D, Van Nieuwenhuizen C, Van Weeghel J (2019). Training professionals in a recovery-oriented methodology: a mixed method evaluation. Scand J Caring Sci.

[35] Bejerholm U, Areberg C, Hofgren C, Sandlund M, Rinaldi M (2015). Individual placement and support in Sweden - a randomized controlled trial. Nord J Psychiatry.

[36] Killaspy H, Priebe S, McPherson P, Zenasni Z, McCrone P, Dowling S (2019). Feasibility randomised trial comparing two forms of mental health supported accommodation (supported housing and floating outreach); a component of the QuEST (quality and effectiveness of supported tenancies) study. Front Psychiatry.

